# Ribosomal proteins: mutant phenotypes by the numbers and associated gene expression changes

**DOI:** 10.1098/rsob.200114

**Published:** 2020-08-19

**Authors:** Michael Polymenis

**Affiliations:** Department of Biochemistry and Biophysics, Texas A&M University, 2128 TAMU, College Station, TX 77843, USA

**Keywords:** yeast, worms, flies, zebrafish, mouse, human

## Abstract

Ribosomal proteins are highly conserved, many universally so among organisms. All ribosomal proteins are structural parts of the same molecular machine, the ribosome. However, when ribosomal proteins are mutated individually, they often lead to distinct and intriguing phenotypes, including specific human pathologies. This review is an attempt to collect and analyse all the reported phenotypes of each ribosomal protein mutant in several eukaryotes (*Saccharomyces cerevisiae*, *Caenorhabditis elegans*, *Drosophila melanogaster*, *Danio rerio*, *Mus musculus*, *Homo sapiens*). These phenotypes were processed with unbiased computational approaches to reveal associations between different phenotypes and the contributions of individual ribosomal protein genes. An overview of gene expression changes in ribosomal protein mutants, with emphasis on ribosome profiling studies, is also presented. The available data point to patterns that may account for most of the observed phenotypes. The information presented here may also inform future studies about the molecular basis of the phenotypes that arise from mutations in ribosomal proteins.

## 1. Overview

Ribosomes are the complex molecular machines that synthesize proteins as instructed from the genetic information on messenger RNAs (mRNAs) [1–4]. Most of the observed phenotypes in cells and organisms arise from the function of polypeptides that the ribosomes produce. Hence, ribosomes are at the critical junction of the genotype-phenotype relation in all species. Fully assembled ribosomes have large and small subunits. The small, and large, subunits in eukaryotes are referred to as the 40S, and 60S subunits, respectively, based on their sedimentation properties. 80S refers to fully assembled ribosomes. Each subunit is a ribonucleoprotein particle, composed of one (in the 40S), or three (in 60S), ribosomal RNA (rRNA) molecules, and many (79 in yeast, 80 in animals) proteins in 80S ribosomes. The ribosomal proteins are structural, non-catalytic components of ribosomes [5,6]. Bacterial ribosomes have similar architecture, but they are smaller and have fewer proteins.

**Figure 1.**
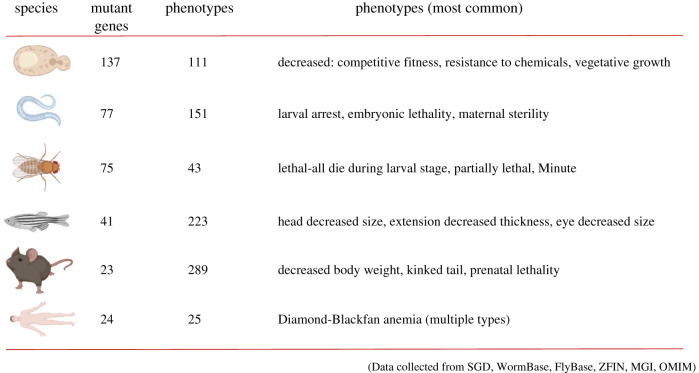
Summary of the most common phenotypes that arise from loss-of-function mutations in ribosomal proteins, in each of the organisms examined in this review. The number of mutant genes for which phenotypes have been described is shown in the second column, while the total number of phenotypes detected by mutations in any ribosomal protein gene in that organism is shown in the third column.

The majority of ribosomal proteins are essential for ribosome function and life. In budding yeast, 15 out of a total of 79 cytoplasmic ribosomal proteins are not essential [7]. In several cases, more than one gene may encode a ribosomal protein. For example, in the budding yeast *Saccharomyces cerevisiae*, 59 ribosomal proteins are encoded in each case by a pair of highly similar paralogous genes. As will be described below (figure 1), cells carrying mutations in ribosomal proteins display a broad spectrum of phenotypes, depending on the locus and alleles involved. It is the objective of this review to systematically go over these phenotypes and examine how they might come about.

The focus in this review is on six eukaryotic organisms, three invertebrate ones (budding yeast, worm, fly) and three vertebrate (fish, mouse, human). The next sections will describe the following: (i) the generation of a complete matrix of ribosomal protein mutant loss-of-function phenotypes in each of the six organisms; (ii) a computational approach to define and group those phenotypes and the genes that underpin them; (iii) a similar analysis for gain-of-function phenotypes of ribosomal proteins in yeast; (iv) a discussion of the evidence linking in some cases ribosomal protein mutants to increased proliferation, including cancer; and (v) an examination of the observed phenotypes in the context of changes in gene expression, especially at the translational level, which may bridge the genotype–phenotype relationships. Lastly, it is worth pointing out that all the datasets generated here are provided in the attached files, with the hope of stimulating further analyses of the remarkable properties and consequences of ribosomal protein perturbations.

## 2. Data input

The gene names of the ribosomal proteins queried are listed in the electronic supplementary material, file S1, in separate spreadsheets for each species. To facilitate comparisons across species, next to each gene name is shown the new unified name of the ribosomal protein that gene encodes [8]. Note that yeast ribosomes lack the eL28 protein. The name of each gene was used to query the well-curated database for each species, to collect all the available phenotypes for that gene in that organism: SGD for *S. cerevisiae* ([9], https://www.yeastgenome.org/); WormBase for *Caenorhabditis elegans* ([10], https://wormbase.org/); FlyBase for *Drosophila melanogaster* ([11], https://flybase.org/); ZFIN for *Danio rerio* ([12], https://zfin.org/); MGI for *Mus musculus* ([13]; http://www.informatics.jax.org/); OMIM for *Homo sapiens* ([14], https://omim.org/). For the most part, primary reports describing ribosomal protein mutant phenotypes were neither cited here nor used as input in the resulting phenotypic matrices. Instead, the collected phenotypes were only those included in each database, with their accompanying descriptors. Because the literature across all these species is expansive, this was the only practical, unbiased, and standardized way to build the phenotypic matrices. Hence, it is possible that additional phenotypes may exist, which were missing in curated databases queried at the time of preparing this review. Nonetheless, even if such missing cases exist, it is unlikely that they would have significantly influenced the outcome of the analyses, because of the large number of data-points already present in the database of each organism.

The phenotypic matrix for each species was assembled from the downloaded individual text files describing the reported phenotypes associated with each gene, as described previously [15]. Each matrix is shown in a sheet (*species*_phenotypes) of a separate supplementary file for each species (e.g. the yeast phenotypic matrix is in the electronic supplementary material, file2/sheet ‘yeast_phenotypes’; the one for worms in the electronic supplementary material, file3/sheet ‘worm_phenotypes’; and so on). For yeast, a separate phenotypic matrix was built for gain-of-function phenotypes, and it will be described separately later in this report.

## 3. General properties of ribosomal protein mutants

An overview of the phenotypes arising from loss-of-function mutations in ribosomal protein genes in all species is in figure 1. The number of the observed phenotypes was considerable, but they were not all observed in most ribosomal protein mutants. With all that information at hand, the first two obvious questions are: what are the most common phenotypes in loss-of-function ribosomal protein mutants, and are there any common patterns across species?

In yeast, 137 genes encoding ribosomal proteins lead to 111 loss-of-function phenotypes (electronic supplementary material, file2/sheet ‘yeast_phenotypes’). The three most common phenotypes in this single-celled organism were decreased competitive fitness, decreased resistance to chemicals, and decreased vegetative growth, observed in 90%, 89% and 80%, respectively, of all the reported loss-of-function mutants. In worms, out of 151 phenotypes observed when 77 loci were mutated, larval arrest, embryonic lethality, and maternal sterility were reported for greater than 84% of all ribosomal protein mutants (electronic supplementary material, file3/sheet ‘worm_phenotypes’). In flies, the most common phenotypes are not shared by as large a portion of mutants as in yeast and worms. Nonetheless, lethality during the larval stage, partially lethality, and the Minute phenotypes were observed in 49%, 40% and 35%, respectively, of all ribosomal protein mutants (electronic supplementary material, file4/sheet ‘fly_phenotypes’). The Minute phenotype has been studied extensively in *Drosophila*, and it has long been recognized to result from cell-autonomous, delayed cell cycle progression and impaired cell growth, leading to smaller cell size [16]. There is a dose-response relationship of the degree of ribosomal protein insufficiency and the strength of the Minute phenotype [17–19]. In zebrafish, there are mutants in about half of the ribosomal protein genes, leading to greater than 200 distinct phenotypes (figure 1). In greater than three-quarters of these mutants, the most common phenotypes were a decreased head size, reduced thickness of the yolk extension, and smaller eyes (electronic supplementary material, file5/sheet ‘fish_phenotypes’). In mice, there are mutants for only 23 ribosomal protein genes (figure 1). Although 289 distinct phenotypes have been observed in these mice, the most common ones, found in approximately one-quarter of these mutants, are decreased body weight, kinked tail and prenatal lethality (electronic supplementary material, file6/sheet ‘mouse_phenotypes’). Viewing this comprehensive data in its totality, it becomes clear that from yeast to mice, the most likely outcomes of loss-of-function mutations in ribosomal protein genes are: reduced or delayed cell proliferation; reduced cell, organ or organismal size; developmental delay, arrest or lethality (figure 1; electronic supplementary material, files 2–6).

In humans, mutations in 24 ribosomal protein genes are linked to disease (figure 1). Patients with mutations in 18 of these loci develop different types of Diamond-Blackfan anemia (electronic supplementary material, file7/sheet ‘human_phenotypes’). The remaining ribosomal protein loci are associated with poor hair cell proliferation (hypotrichosis), poor bone growth (leading to dysplasias and short stature), shorter skull (brachycephaly), absence of a spleen (asplenia), developmental delay, refractory macrocytic anemia, mental retardation, or autism. Although ribosomal protein mutations are associated with distinct types of Diamond-Blackfan anemia, in all cases, there is a failure of the bone marrow to develop properly and produce enough red blood cells [20]. There are also additional abnormalities [20], which are consistent with the most common phenotypes observed in the other model systems discussed above. For example, about half of the Diamond-Blackfan patients have physical abnormalities. These abnormalities are manifested as an unusually small head (microcephaly), small lower jaw (micrognathia) and other malformations. About a third of affected individuals also grow slowly and have short stature. Hence, in humans, as in the different organisms discussed above, the typical phenotypic manifestations of ribosomal protein loss-of-function mutations are, in essence, consequences of hypo-proliferation.

But as satisfying as the congruence of the most common phenotypes of ribosomal protein mutants may be from yeast to humans, this view oversimplifies the underlying biology. It would be erroneous to conclude that ‘you‘ve seen one ribosomal protein mutant, you‘ve seen them all’. After all, there is still such a broad spectrum of additional phenotypes in each organism (electronic supplementary material, files 2–7). The apparent multitude of these phenotypes raises further questions, such as: to reduce this complexity, can one identify phenotypes that cluster together in different groups? If so, what are the ribosomal protein genes that drive this classification? Answering these questions may offer new insights into phenotype–phenotype and gene-phenotype associations among ribosomal protein mutants.

## 4. Multiple correspondence analysis of ribosomal protein phenotypes

Treating the different phenotypes as distinct variables, one could apply widely used multivariate statistical techniques to simplify related phenotypic variables. Measuring the degree that the observed phenotypic variables correlate with each other, provides the basis for reducing them. If two or more phenotypic variables share some features, then based on the magnitude and direction of the relationship, the observed complexity may be simplified. Techniques implementing the above principles include factor analysis and principal component analysis [21]. For categorical data (e.g. the presence or absence of a phenotype), a related approach is that of correspondence analysis [22], to detect and group underlying structures in the phenotypic variables within a dataset [15]. As a result, one obtains a lower-dimensional view of the internal structure of the data. Such approaches can be applied to datasets where each mutant displays at least a few phenotypes. This is the case for the ribosomal protein mutants in the model organisms we discussed above, except in humans. The phenotypic terms associated with almost all ribosomal protein mutants in humans are unique to each mutant. For *RPL10*, there are two associated diseases: autism and spondyloepimetaphyseal dysplasia (electronic supplementary material, file7/sheet ‘human_phenotypes’). Note that although anemias are prevalent among ribosomal protein mutant patients, each locus leads to a unique type of Diamond-Blackfan anemia (electronic supplementary material, file7/sheet ‘human_phenotypes’), which were kept as separate phenotypic variables. Hence, there is a near one-to-one correspondence between a phenotypic variable and ribosomal protein locus, which precludes any attempt to reduce the dimensionality of the human dataset.

**Figure 2.**
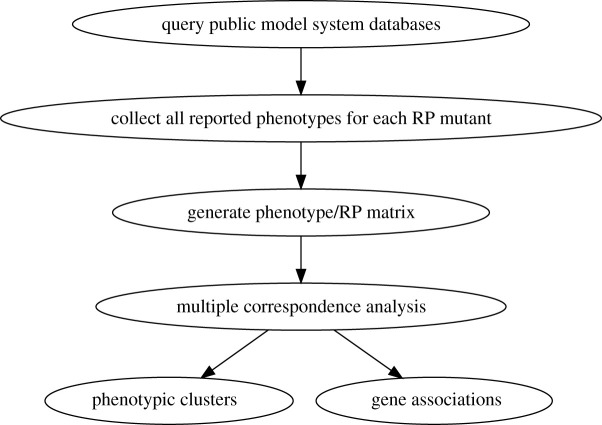
Schematic of the process to reduce the complexity of the observed phenotypes among ribosomal protein (RP) mutants and identify the genes that contribute the most to specific groups.

**Figure 3.**
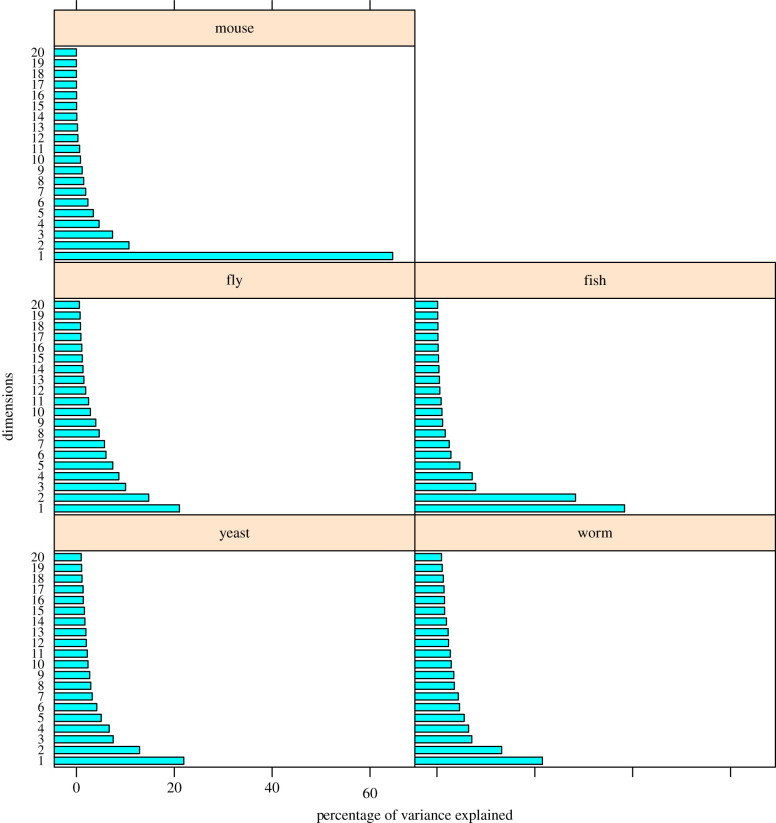
Scree plots for the first 20 dimensions in each species, showing the percentage of the variance explained by each dimension in each organism. For the full list of all the dimensions, see the electronic supplementary material file for each organism, in the sheets denoted ‘*_eigen’.

**Figure 4.**
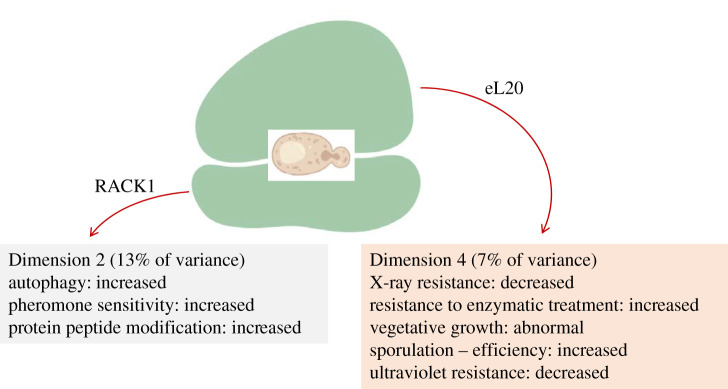
Phenotypes that show the most significant association with specific dimensions/clusters among loss-of-function ribosomal protein mutants in yeast. The ribosomal proteins that drive these groupings are indicated in each case.

**Figure 5.**
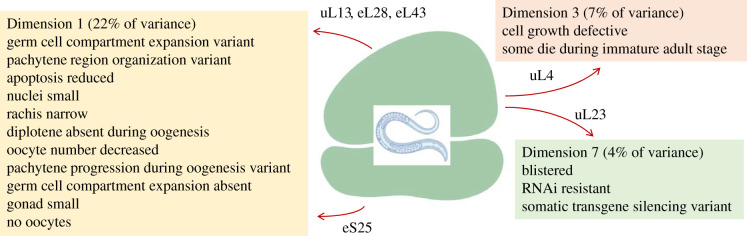
Phenotypes that show the most significant association with specific dimensions/clusters among loss-of-function ribosomal protein mutants in worms. The ribosomal proteins that drive these groupings are indicated in each case. All the proteins shown had significant contributions (correlation coefficients > 0.4).

**Figure 6.**
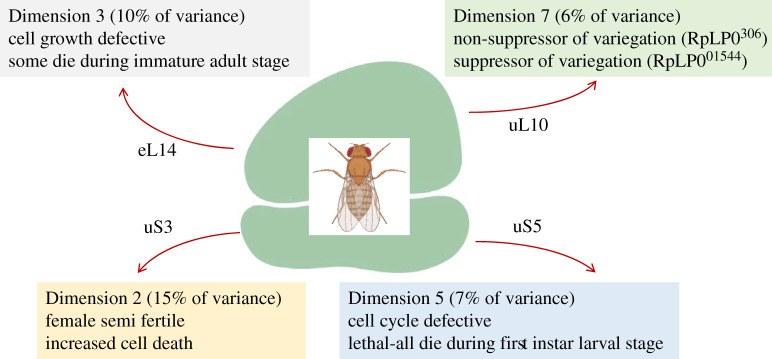
Phenotypes that show the most significant association with specific dimensions/clusters among loss-of-function ribosomal protein mutants in flies. The ribosomal proteins that drive these groupings are indicated in each case.

**Figure 7.**
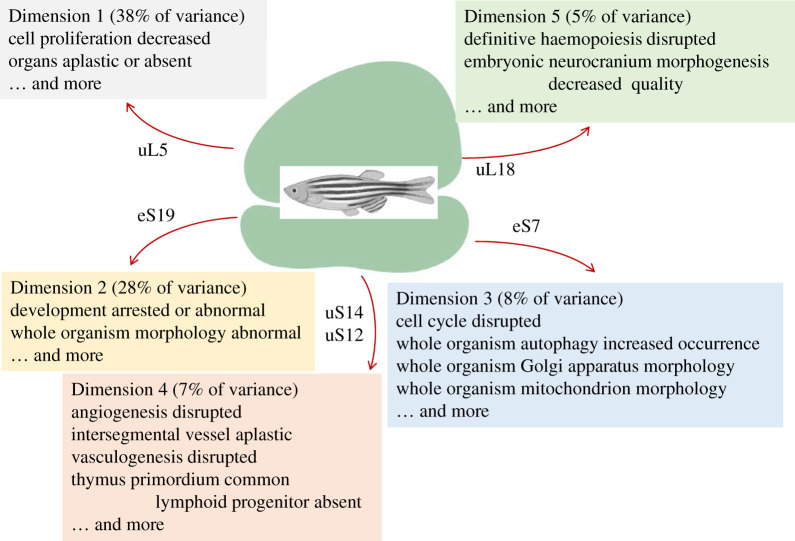
Phenotypes that show the most significant association with specific dimensions/clusters among loss-of-function ribosomal protein mutants in zebrafish. The ribosomal proteins that drive these groupings are indicated in each case. All the proteins shown had significant contributions (correlation coefficients >0.4).

**Figure 8.**
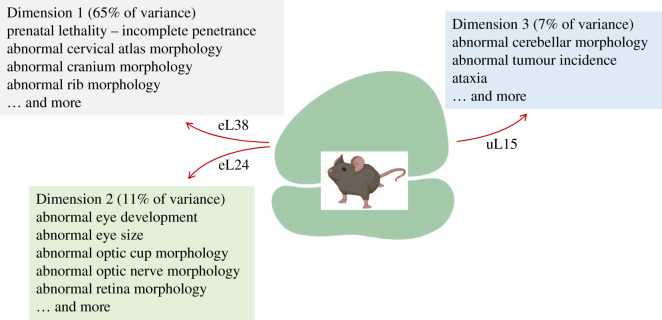
Phenotypes that show the most significant association with specific dimensions/clusters among loss-of-function ribosomal protein mutants in mice. The ribosomal proteins that drive these groupings are indicated in each case.

**Figure 9.**
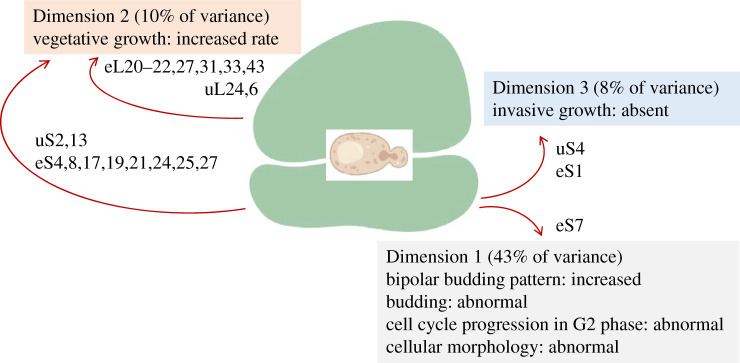
Phenotypes that show the most significant association with specific dimensions/clusters among gain-of-function ribosomal protein mutants in yeast. The ribosomal proteins that drive these groupings are indicated in each case. All the proteins shown had significant contributions (correlation coefficients >0.4).

For the ribosomal protein mutant phenotypes for each of the other species, multiple correspondence analysis (MCA) was performed as described elsewhere [15]. The process is summarized in figure 2. The percentage of the variance by the first 20 dimensions in each species is shown in the scree plots in figure 3. In the next paragraphs, the following will be described for each species: (i) the number of the dimensions/clusters that explain most of the variance in the observed phenotypes; (ii) the phenotypes that contribute the most to each dimension (discussion will be limited to those phenotypes with an arbitrarily chosen cutoff of correlation ≥ 0.4); and (iii) the individual genes contributing the most to each dimension (again, the discussion will be limited to genes with correlations ≥ 0.4). All the data for each organism can be found in the corresponding supplementary files. Separate displays (figures 4–9) for each organism show the dimensions that were significantly driven both by specific phenotypic variables *and* by specific ribosomal protein genes (i.e. correlations greater than 0.4 in both cases). Overall, this approach might offer valuable insight about the variance in the data and reduce the bewildering complexity of ribosomal protein mutant phenotypes.

### 4.1. Saccharomyces cerevisiae

In yeast, the 111 loss-of-function phenotypes could be reduced to 11 dimensions. Together, these 11 dimensions accounted for 72% of the variance in the phenotypic variables (electronic supplementary material, file2/sheet ‘yeast_eigen’). In most of the dimensions (listed in the electronic supplementary material, file2/sheets ‘yeast_Dim’), the associated phenotypes were broadly dispersed and did not strongly associate with a given dimension (i.e. the correlation coefficients were less than 0.4). Note that the most common phenotypes in this organism (e.g. reduced fitness; figure 1) are displayed in greater than 80% of the ribosomal protein mutants. Nonetheless, we noted that increased autophagy and sensitivity to pheromone were significantly related to Dimension 2 (*R*^2^ = 0.58; see the electronic supplementary material, file2/sheet ‘yeast_Dim2’). Dimension 2 accounts for 13% of the variance among all 111 phenotypes. Increased sensitivity to pheromone probably reflects the prolonged G1 phase [23] observed in ribosomal protein mutants [24]. Autophagy is a strategy to obtain the resources necessary to sustain some degree of proliferation when nutrients are limiting, or during other stresses [25]. Hence, it is reasonable to expect increased autophagy in ribosomal protein perturbations, which may genetically mirror a nutrient-poor, stress environment.

A valuable outcome of the multiple correspondence analysis outlined above is pointing to the mutant gene that drives the grouping of the various phenotypic variables in each dimension (listed in the electronic supplementary material, file2/sheet ‘yeast_genes_cos2’). Interestingly, mutations in the ribosomal protein Asc1p (RACK1 in the unified nomenclature) drives the grouping in Dimension 2, dominated by increased autophagy and pheromone sensitivity (figure 4). Asc1p/RACK1 prevents frameshifting in paused ribosomes [26]. Ribosome pausing often occurs when the supply of amino acids is limited [27]. The inability of ribosomes to properly pause, in cells lacking RACK1, may mimic conditions that induce autophagy.

Reduced resistance to X-rays was strongly associated with Dimension 4 (*R*^2^ = 0.69; see the electronic supplementary material, file2/sheet ‘yeast_Dim4’). This dimension only accounts for 7% of the variance among all the ribosomal protein mutant phenotypes (electronic supplementary material, file2/sheet ‘yeast_eigen’), and mutations in eL20 drove that grouping (figure 4). Interestingly, however, this contribution was paralogue-specific (electronic supplementary material, file2/sheet ‘yeast_genes_cos2’), from *RPL20A* (*R*^2^ = 0.77), but not from *RPL20B* (*R*^2^ = 0.0009). We will return to the issue of paralogue-specific phenotypes later.

### 4.2. Caenorhabditis elegans

In worms, the 151 loss-of-function phenotypes were also reduced to 11 dimensions, accounting for 76% of the variance (electronic supplementary material, file3/sheet ‘worm_eigen’). At least three of the dimensions were driven strongly by specific phenotypes (figure 5). For example, the first dimension in this metazoan organism, accounting for 22% of the variance among all the phenotypic variables, is a mix of cellular, tissue, and organismal manifestations of hypo-proliferation, including small cells and nuclei, small or absent tissues, and a narrowing of the central body axis (figure 5; electronic supplementary material, file3/sheet ‘worm_Dim1’). Lastly, the ribosomal protein genes that were most significantly associated with these phenotypic groups (figure 5), encoded mostly proteins of the large ribosomal subunit (uL13, eL28, eL43, uL4, uL23; electronic supplementary material, file3/sheet ‘worm_genes_cos2’).

### 4.3. Drosophila melanogaster

In flies, grouping the 43 loss-of-function phenotypes into eight dimensions explained 78% of the variance (electronic supplementary material, file4/sheet ‘fly_eigen’). As in worms, hypo-proliferative manifestations, such as defective cell growth, cell cycle, reduced fertility, or lethality, dominated the different groups (figure 6). The exception was Dimension 7, accounting for 6% of the total phenotypic variance, which was dominated by the ability of some, but not all, uL10 alleles to suppress variegation (electronic supplementary material, file4/sheet ‘fly_Dim7’). It is worth noting that cell cycle defects dominated Dimension 5, driven by a RpS2/uS5 mutant (electronic supplementary material, file4/sheet ‘fly_Dim5’).

### 4.4. Danio rerio

In fish, the number of phenotypic variables observed in ribosomal protein mutants expands significantly (figure 1), reflecting the added complexity of vertebrate biology. Remarkably, however, all these phenotypes could be reduced to just five dimensions, capturing 86% of the variance (electronic supplementary material, file5/sheet ‘fish_eigen’). A detailed list of the phenotypes and genes that are most significantly associated with each dimension is in the electronic supplementary material, file5. They are also summarized schematically in figure 7. The typical hypo-proliferative phenotypes displayed in the other model systems discussed so far, are also evident in fish.

Moreover, disrupted definitive haematopoiesis and defective neurocranium morphogenesis were closely associated with Dimension 5 (figure 7; electronic supplementary material, file5/sheet ‘fish_Dim5’). As discussed above, these are phenotypes also seen in human patients with Diamond-Blackfan anemias (electronic supplementary material, file7/sheet ‘human_phenotypes’). The gene driving this grouping in fish is *rpl5a*/uL18 (electronic supplementary material, file5/sheet ‘fish_Dim5’). Mutations in the human orthologue, *RPL5*/uL18, lead to Diamond-Blackfan anemia type 6. Lastly, it is worth pointing out that at the cellular level, increased autophagy was significantly associated with Dimension 3 in fish (figure 7), as was seen for one of the dimensions in yeast (figure 3). Overall, the above observations offer remarkable examples of the conservation of the phenotypic manifestations of ribosomal protein perturbations across multiple species.

### 4.5. Mus musculus

In mice, there are 23 reported ribosomal protein mutants, displaying an astonishing 289 distinct phenotypic variables (figure 1; electronic supplementary material, file6/sheet ‘mouse_phenotypes’). However, all these phenotypes could be grouped in just three dimensions, explaining 83% of the observed variance (electronic supplementary material, file6/sheet ‘mouse_eigen’). The phenotypes and genes that are most significantly associated with each dimension are in the electronic supplementary material, file6 and shown schematically in figure 8. As discussed above for fish and humans, skeletal abnormalities are also prominent in mouse ribosomal protein mutants.

## 5. Gain-of-function phenotypes of ribosomal proteins in yeast

In yeast, there are 24 reported phenotypes associated with the over-expression of 75 ribosomal protein genes (electronic supplementary material, file2/sheet ‘yeast_gof_phenotypes'). The most common phenotypes were changes in the rate of vegetative growth, which *increased* for 32 genes but *decreased* for 19 others. For four genes (*RPL24B*, *RPL34A*, *RPL37B*, *RPS22B*), there were conflicting reports that vegetative growth was either increased or decreased (electronic supplementary material, file2/sheet ‘yeast_gof_phenotypes’). The 24 phenotypes associated with the over-expression of ribosomal proteins could be grouped in three dimensions, explaining 61% of the observed variance (electronic supplementary material, file2/sheet ‘yeast_gof_eigen’). Dimension 1, accounting for 43% of the total variance, is driven by abnormal morphology and cell cycle progression in G2. Yeast displays characteristic patterns of polarized growth and budding when it proliferates, which were affected by ectopic ribosomal protein expression, especially of RPS7A/eS7 (figure 9; electronic supplementary material, file2/sheet ‘yeast_gof_genes_cos2’). Numerous genes, encoding proteins of both the large and small ribosomal subunits, contributed to Dimension 2, characterized by increased vegetative growth (figure 9). Invasive growth in yeast is also associated with polarized growth [28]. The absence of invasive growth drove the grouping in Dimension 3 (figure 9). Hence, there appears to be a general pattern of altered polarized growth when ribosomal proteins are over-expressed in yeast.

## 6. Over-proliferation in ribosomal protein mutants

The increased proliferation observed when at least some ribosomal proteins are over-expressed in yeast is intriguing, but also puzzling. It is not known if those effects are reflections of ribosomal output, or of some unknown, extra-ribosomal function. Even if the increased cell proliferation is associated with ribosomal functions and more protein synthesis, it is unclear how over-expression of a single component of a giant molecular machine made of many parts, could drive the formation of more such machines. However, a recent report in mice argued that over-expression of *RPL15* (eL15) not only enhanced translation of other genes, including cell cycle regulators, but also promoted distant metastases in mice with breast cancer [29].

Increased proliferation and cancer have also been associated with loss-of-function ribosomal protein mutations. As discussed above, early in life ribosomopathies are consistent with hypo-proliferation, such as defective haematopoiesis in Diamond-Blackfan anemias [30]. Paradoxically, later in life, some of these patients are predisposed to cancer [30,31]. Ten per cent of primary human samples of T-cell acute lymphoblastic leukemia have loss-of-function mutations in *RPL22*/eL22 [32]. *RPL22* mutations are also found in microsatellite-unstable colorectal [33], and endometrial cancers [33,34], at 77%, and 50% frequency, respectively. In addition, cancer-associated mutations have been described for *RPL5* (uL18) [35], *RPL10* (uL16) [35], *RPL11* (uL5) [36], *RPS15* (uS19) [37,38], *RPS20* (uS10) [39] and *RPS14* (uS11) [40,41]. The cancer-associated mutations in ribosomal proteins are hypomorphic ones, impairing ribosome biogenesis [30]. Even a missense R98S mutation in *RPL10* (uL16) observed in T-cell leukemia, was shown to impair ribosome biogenesis and delay cell proliferation when introduced in yeast and mammalian cells [35]. Evidence that ribosomal proteins may function as haploinsufficient tumour suppressors has been reported in zebrafish [42] and flies [43–45]. However, these results do not necessarily support a direct, negative role of ribosome biogenesis in cell division. Indeed, such effects in flies were owing to cell non-autonomous routes [46–48]. Overall, in the context of their role in protein synthesis, most of the evidence suggests that the initial phenotype upon loss-of-function perturbations of ribosomal proteins is hypo-proliferative. How then could ribosomal protein perturbations account for uncontrolled cell proliferation in cancer? There are at least three possibilities, which are not exclusive of each other:

Ribosomal protein perturbations reduce the *concentration* of active ribosomes [49–51], which then *disproportionately* affects translation of specific transcripts [51,52]. Ribosomal proteins themselves may not be direct negative regulators of cell division, but in ribosomal protein mutants, translation of some mRNAs, perhaps some with tumour suppressor roles, could be repressed more so than other transcripts, setting the stage for cancer. The mathematical background for this type of regulation was articulated long ago by Lodish [53]. Briefly, the Lodish model predicts mRNA-specific effects because of the nonlinear relationship between translational efficiency and the available ribosomes. In decreasing ribosome content, e.g. upon perturbations of ribosomal proteins in ribosomopathies, mRNAs with features (e.g. secondary structure, upstream open reading frames) that impede ribosome access to the main start codon of an mRNA will have a *disproportionately* lower translational efficiency than other mRNAs. The proposition that mRNA-specific cases of translational control, as predicted by the Lodish model, may underpin at least some of the phenotypes in ribosomopathies [51,52], is reasonable and straightforward.

Ribosomal proteins could have *extra-ribosomal*, non-translational functions [54,55]. Disruption of ribosome biogenesis induces nucleolar stress because free ribosomal proteins accumulate. Loss of Rpl22 may lead to cancer in mice by activating the stress-induced NF-κB pathway, which in turn triggers the stemness factor Lin28B [32]. When ribosome assembly is disrupted, some of the released ribosomal proteins could bind other targets. For example, Rpl5, Rpl11 and Rpl23 have been reported to stabilize the p53 protein, by inhibiting the Mdm2 ubiquitin ligase that degrades p53 [55]. It is not clear, however, how this extra-ribosomal role could promote cancer, as stabilization of the p53 tumour suppressor would probably be *hypo*-proliferative. A recent study in human cells lacking Rps25/eS25 reported that cellular adaptation to ribosomal protein loss, rather than direct translation control, can drive phenotypes assumed to result from preferential translation [56]. In that scenario, upon eS25 loss, the cellular ribosome pool was under a stress relating to its biogenesis and turnover, eliciting a specific cellular state change, which itself drives phenotypes [56].

Lastly, impairing ribosomal proteins could alter the *composition* of active ribosomes [57,58]. Translation of mRNAs that rely on ‘specialized’ ribosomes has been reported, especially in neurons [59]. However, there are no examples of transcripts whose translation is carried out by ‘specialized’ ribosomes *and* affected in cancers owing to ribosomal protein perturbations.

Regardless of the validity of each of the above models, until recently, there was very little information about gene expression changes in ribosomal protein mutants and, specifically, about the translational efficiency of *all* mRNAs in those settings. Without such knowledge, it is difficult to bridge the genotype-phenotype relationship in ribosomal protein mutants mechanistically. However, in the last 2–3 years, some answers have emerged, based on recent findings from ribosome profiling in ribosomal protein mutants, which will be discussed in the next section.

## 7. Gene expression changes in ribosomal protein mutants

Before discussing ribosome profiling experiments in ribosomal protein mutants, it should be noted that a few changes in the expression of specific gene products in some of those mutants have been catalogued in zebrafish. These data are in the electronic supplementary material, file5/sheet ‘fish_gene_expression’. It covers the reported changes at the levels of 36 loci in three mutants (*rpl11*, *rpl5a*, *rps19*), which may offer some insight into the phenotypes observed. In these cases, however, how the changes in gene expression came about was not clear.

Ribosome profiling incorporates next-generation sequencing to quantify all the pieces of mRNAs bound to ribosomes [60–62]. From the accompanying RNAseq data, for each mRNA species, one can compute from the observed steady-state levels of that mRNA as a reference, if the fraction that is bound to ribosomes is higher than expected, or lower, indicating an increased, or decreased, translational efficiency, respectively. In human cells, Khajuria and colleagues mimicked a Diamond-Blackfan setting by suppressing *RPS19* (eS19), *RPL5* (uL18), *RPS24* (eS24) and *RPL11* (uL5) [51]. In all cases, haematopoietic cells had lower levels of ribosomes, but the composition of the ribosomes did not change. The consequences of *RPL5* and *RPS19* suppression were then analysed by ribosome profiling. Changes in transcription and translation were similar between *RPL5* and *RPS19* mutants, arguing that Diamond-Blackfan anemias lead to a common set of molecular changes in human haematopoietic cells. Importantly, translation of a subset of transcripts that are normally upregulated at the early stages of erythroid lineage-specification, including *GATA1*—which encodes a transcription factor that triggers the differentiation of immature blood cells, was disproportionately reduced when *RPL5* and *RPS19* were repressed [51]. Translation of mRNAs encoding ribosomal proteins was also lower in these settings [51].

A similar general conclusion that lower ribosome levels result in specific and dose-dependent changes in gene expression was also reached by an elegant study in yeast [50]. These authors analysed by ribosome profiling 14 *rpl* and 9 *rps* mutants, each lacking one of the paralogues that encode the corresponding ribosomal protein. The primary phenotypic readout used in that study was the rate of vegetative growth. Remarkably, the patterns of gene expression changes matched the growth rate of each mutant [50]. In other words, if an *rpl* and an *rps* deletion have a similar effect on the growth rate, then the associated gene expression changes would also be similar. Unlike the situation in human cells, Cheng and colleagues found that the translation of genes involved in ribosome biogenesis was increased (not decreased), especially in *rps* mutants [50].

Besides general effects on the growth rate, more nuanced and specific effects must also exist, for several reasons. First, the spectrum of the phenotypes observed in ribosomal protein mutants is varied and complex. Second, the growth rate is a simple, quantitative parameter, but using growth rate changes alone as a criterion to evaluate ribosomal protein phenotypes runs the risk of ‘missing the trees for the forest’. Different cellular pathways may be affected by different ribosomal protein mutants, but these different inputs may be missed if they have comparable impacts on growth rate. For example, some ribosomal protein mutants often exhibit an equivalent G1 cell cycle delay, but for different reasons [63]. At least some phenotypes strongly associated with ribosomal protein mutations do not correlate at all with dose-dependent effects on growth rate. Such an example is replicative longevity. Mutations in ribosomal proteins of the large (60S) subunit promote longevity in yeast [7,49,64,65]. The relationship between *rpl* mutants and longevity is complex. For example, the Rpl22 double paralogue deletion is viable, but not long-lived [7]. The single *rpl22aΔ* mutant is long-lived, but *rpl22bΔ* cells are not long-lived [7], and there is no relationship between the growth rate of *rpl* mutants and their longevity [66].

Yet another demonstration of the power of ribosome profiling to provide the mechanistic underpinning of translational effects and their phenotypic consequences comes from studies that examined paralogue pairs in yeast, including the Rpl22 pair [66]. The authors found a small set (less than 100) of mRNAs that were differentially translated. These mRNAs were significantly enriched for transcripts that encode enzymes of one-carbon metabolism. Metabolomic measurements supported the conclusion that one-carbon metabolism is specifically downregulated in cells lacking Rpl22Ap, but not Rpl22B, accounting for all the phenotypes of *rpl22aΔ* cells, including in longevity [66]. As in the previous studies mentioned above [50,51], there was no change in bulk ribosome composition in *rpl22* mutants [66]. In agreement with Cheng *et al*. [50], compared to wild-type cells, translation of transcripts encoding other ribosomal proteins was increased in the paralogue deletants, even though overall protein synthesis was reduced [66]. It seems that yeast cells attempt to offset their reduced protein synthesis capacity by increasing the levels of individual components of the ribosome. But these efforts do not globally restore the protein synthesis defect, presumably because the production of ribosomal components is unbalanced.

## 8. Concluding remarks

The general picture that emerges from the detailed profiling studies is straightforward: loss-of-function ribosomal protein mutants → fewer ribosomes → lower protein synthesis → general hypo-proliferation and dose-dependent, disproportionate translational control of a subset of mRNAs. This is a broad view that corresponds very well with the most common phenotypes summarized earlier from yeast to humans (figure 1). Additional, more specific effects that are uncoupled from the growth rate can also be accounted for by translational control of relevant transcripts [66]. The stress associated with the lower ribosome pool in ribosomal protein mutants may also trigger secondary changes, leading to stress-associated phenotypes, with no direct translational basis [56]. Nonetheless, from the evidence collected thus far, it appears that the varied phenotypic landscape of ribosomal protein mutants, from the general to more peculiar phenotypes, mainly comes about from the canonical roles of ribosomal proteins in ribosomes. The profiling studies did not support additional mechanisms of specialized ribosomes with altered composition or extra-ribosomal functions, but it was also not explicitly evaluated. Hence, these conclusions need to be tested further and in more detail. Applying these methodologies to the analysis of more ribosomal protein mutants that display phenotypes of interest, will undoubtedly advance our knowledge in the relationship between genotype and phenotype in ribosomal protein perturbations, illuminating their fascinating biology and the broader roles of translational control.

## Supplementary Material

Yeast MCA datasets

## Supplementary Material

Worm MCA datasets

## Supplementary Material

Fly MCA datasets

## Supplementary Material

Ribosomal Proteins and Genes

## Supplementary Material

Fish MCA datasets

## Supplementary Material

Mouse MCA datasets

## Supplementary Material

Human ribosomal protein phenotypes

## Data accessibility

All the data are provided as the electronic supplementary material.
